# Evaluating the Role of Multimodal Imaging in the Diagnosis of Cardiac Amyloidosis and Hypertrophic Cardiomyopathy

**DOI:** 10.1111/anec.70086

**Published:** 2025-05-10

**Authors:** Zi‐xin Yang, Rong‐hui Zheng, Cui‐yan Wang, Hai‐tao Yuan, Yong‐le Sun, Mei Zhu

**Affiliations:** ^1^ Department of Cardiovascular Ultrasonography Central Hospital Affiliated to Shandong First Medical University Jinan China; ^2^ Department of Ultrasonography Shandong Provincial Hospital Affiliated to Shandong First Medical University Jinan China; ^3^ Department of Medical Imaging Shandong Provincial Hospital Affiliated to Shandong First Medical University Jinan China; ^4^ Department of Cardiology Shandong Provincial Hospital Affiliated to Shandong First Medical University Jinan China

**Keywords:** cardiac amyloidosis (CA), cardiac magnetic resonance (CMR), echocardiography, hypertrophic cardiomyopathy (HCM), multimodal imaging

## Abstract

**Objective:**

The objective of this study is to examine the evolving cardiac characteristics of patients with cardiac amyloidosis (CA) and hypertrophic cardiomyopathy (HCM) by integrating multimodal imaging techniques, including conventional echocardiography, strain echocardiography, and cardiac magnetic resonance imaging.

**Methods:**

A retrospective study was conducted, comprising 38 patients with CA, 20 patients with HCM, and 16 healthy individuals in the control group. Statistical analyses were conducted to assess conventional and strain echocardiography parameters across these groups. Furthermore, cardiac magnetic resonance imaging data from 15 patients with CA and 15 patients with HCM were analyzed and compared, focusing on correlations between imaging parameters and myocardial amyloid load.

**Results:**

Analysis of conventional and strain echocardiography revealed that left ventricular ejection fraction, E/e′, relative apical longitudinal sparing, and the ejection fraction‐to‐longitudinal strain ratio were strongly associated with the diagnosis of CA and served as key differentiators between the CA and HCM groups. The combination of these four parameters yielded optimal diagnostic efficiency, with an area under the curve of 0.916.

**Conclusion:**

The integration of conventional and strain multiparametric echocardiography demonstrated superior diagnostic efficacy in differentiating CA from HCM. Furthermore, the analysis of cardiac magnetic resonance parameters indicated that an increase in cardiac amyloid load is associated with changes in cardiac indices, with parameters such as E/e′, basal longitudinal strain, global longitudinal strain, and ejection fraction‐to‐strain ratio effectively reflecting the extent of amyloid infiltration in the myocardium.

## Graphical abstract

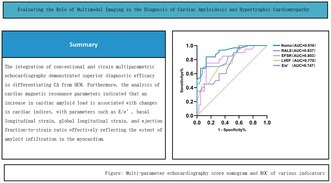

**Abbreviations**
AL‐CA: light chain amyloidosis
ATTR‐CA: transthyretin cardiac amyloidosis
AUC: area under the curve
CA: cardiac amyloidosis
ECV: extracellular volume
ESC: European Society of Cardiology
GLS: global longitudinal strain
HCM: hypertrophic cardiomyopathy
ICC: intraclass correlation coefficient
IVSd: interventricular septal thickness at diastole
LAD: left atrial diameter
LS: longitudinal strain
LVEDD: left ventricular end diastolic dimension
LVEF: left ventricular ejection fraction
LVPWd: left ventricular posterior wall thickness at diastole
ROC: receiver operating characteristic
RWT: relative wall thickness

## 1 Introduction

Cardiac amyloidosis (CA) is characterized by the accumulation of misfolded amyloid fibrils within cardiac tissue (Lachmann and Hawkins 2006). The two most prevalent subtypes are systemic immunoglobulin light chain cardiac amyloidosis (AL‐CA) and transthyretin cardiac amyloidosis (ATTR‐CA). The accumulation of amyloid fibrils in CA results in pathological changes such as impaired left ventricular diastolic function, arrhythmias, myocardial fibrosis, myocardial ischemia, heart failure, and sudden cardiac death, all of which significantly contribute to patient mortality (Wang et al. 2023).

Hypertrophic cardiomyopathy (HCM), while more prevalent than CA, exhibits notable diagnostic challenges due to a disparity between its estimated prevalence and the frequency of clinical diagnoses, highlighting potential diagnostic gaps (Gillmore et al. 2018). Patients with HCM and CA often present with overlapping clinical symptoms and imaging features, making differential diagnosis challenging (Kittleson et al. 2020). However, the treatment methods for CA and HCM differ significantly, emphasizing the critical need for early detection of CA and accurate differentiation from HCM to optimize patient management.

Automated Cardiac Motion Quantification (aCMQ) technology, derived from speckle tracking imaging, is a two‐dimensional strain imaging technique that enables automated quantification and comprehensive analysis of the entire myocardium. This technology allows for the detection of early changes in left ventricular systolic function in patients with CA and HCM (Garcia‐Pavia et al. 2021; Schiano‐Lomoriello et al. 2016).

Along with conventional and strain echocardiography, advanced cardiac magnetic resonance (CMR) techniques, including T1 mapping and late gadolinium enhancement, provide valuable insights into myocardial pathology. These CMR modalities are capable of identifying myocardial fibrosis and amyloid deposition within the extracellular matrix of myocardial cells, reflecting the extent of amyloid burden on the heart (Dohy et al. 2022; Pan et al. 2020). Such imaging tools offer significant diagnostic advantages and contribute to the differentiation of CA from HCM.

Most studies have focused on examining the impact of amyloid infiltration on the functional performance of the left and right heart chambers by using echocardiography and CMR imaging across various levels. However, there have been only a few studies that have explored the correlation between echocardiographic and CMR parameters that reflect cardiac amyloid burden. This study used a multiparametric echocardiographic scoring approach to differentiate between CA and HCM. Furthermore, it integrated echocardiographic and CMR imaging parameters to provide a theoretical foundation for the early diagnosis and accurate differentiation of patients with CA.

## 2 Material and Methods

### 2.1 General Data

From January 2018 to November 2023, a cohort of 38 patients meeting the diagnostic criteria for CA was identified, including 27 patients with AL‐CA and 11 with ATTR‐CA. These patients were selected from the Shandong Provincial Hospital affiliated with Shandong First Medical University, based on specified inclusion and exclusion criteria. Among these patients, 15 underwent CMR imaging. Myocardial or extracardiac biopsies were conducted on all patients, confirming amyloid deposition with positive Congo red staining. Additionally, 20 patients with HCM and 16 healthy individuals attending routine outpatient check‐ups were included as the control group. Ethical approval for the study was granted by the Ethical Academic Committee of the hospital (Approval No.: SWYX: NO. 2022‐378).

### 2.2 Inclusion Criteria

Inclusion criteria for the CA Group: Patients were included if they were diagnosed with CA based on clinical evaluations, pathological biopsy results, or imaging findings. Additionally, echocardiographic evidence of left ventricular wall thickening (defined as an interventricular septum or posterior wall thickness of ≥ 12 mm) was required.

Inclusion criteria for the HCM Group: Patients were included if echocardiography demonstrated a septal or left ventricular wall thickness of at least 15 mm without significant obstruction in the left ventricular outflow tract. For patients with a definitive family history of HCM or positive genetic testing, a threshold of at least 13 mm was applied, provided that other potential causes of increased ventricular wall thickness, such as elevated cardiac load, were excluded.

### 2.3 Exclusion Criteria

1. Presence of comorbid cardiomyopathies associated with infiltration or increased cardiac load, such as hypertensive cardiomyopathy, sarcoidosis, or diabetic cardiomyopathy.
2. Insufficient quality of dynamic imaging data renders accurate myocardial identification by the analytical software unattainable.
3. Coronary artery stenosis > 50%, or a history of coronary stent implantation or coronary artery bypass grafting.
4. Severe hepatic or renal dysfunction, or the presence of malignant tumors.

### 2.4 Instruments and Methods

#### 2.4.1 Instruments

Ultrasound echocardiography images were acquired by various operators using the EPIQ 7C Doppler ultrasound diagnostic system (Philips, USA), with an image frame rate of ≥ 50 FPS. The S5‐1 cardiac probe was used, operating at a frequency range of 1.0 to 5.0 MHz. Strain analysis was conducted on DICOM‐format images using aCMQ software (Qlab 13.0 version).

CMR images were obtained using 3.0 T magnetic resonance scanners (Achieva, Philips; Skyra, Siemens). Quantitative assessments of longitudinal relaxation time and extracellular volume were conducted on DICOM‐format images using cvi42 software.

#### 2.4.2 Image Collection and Data Measurement

##### 2.4.2.1 Clinical Information Collection

Patient gender, age, presenting symptoms, physical signs, body surface area, admission blood pressure, and laboratory indicators such as N‐terminal pro‐B‐type natriuretic peptide (NT‐proBNP) and high‐sensitivity troponin T (HS‐TnT). The collected clinical characteristics were systematically collated for further analysis.

##### 2.4.2.2 Conventional Echocardiography Parameters

Conventional echocardiographic assessments, including measurements of atrial and ventricular dimensions, evaluations of left ventricular systolic and diastolic function, and Doppler parameters, were conducted in accordance with the latest guidelines established by professional societies. Measurements included left atrial diameter (LAD), left ventricular end‐diastolic dimension (LVEDD), interventricular septal thickness at diastole (IVSd), left ventricular posterior wall thickness at diastole (LVPWd), left ventricular ejection fraction (LVEF), mitral valve forward flow pulse Doppler spectral peaks (E and A), and septal and lateral wall tissue Doppler spectral e′ velocities. Additionally, calculations were conducted and recorded for relative wall thickness (RWT), the mitral forward flow velocity ratio (E/A), and the ratio of mitral diastolic flow spectrum to tissue spectrum (E/e′).

##### 2.4.2.3 Strain Echocardiography Parameters

**FIGURE 1:**
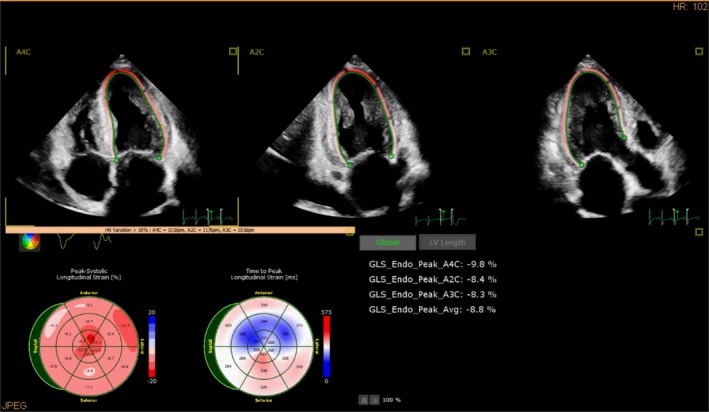
Myocardial longitudinal strain value and 18‐segment bull's eye map of the left ventricular regions in patients with CA.

The speckle‐tracking method, applied to images in DICOM format and analyzed using aCMQ software, was used to automatically identify and calculate myocardial longitudinal strain (LS) and left ventricular global longitudinal strain (GLS) across the apical four‐chamber, two‐chamber, and three‐chamber views, focusing primarily on left ventricular endocardial strain. By using the 18‐segment bull's‐eye plot, the average myocardial LS values for the apical (apiLS), middle (medLS), and basal longitudinal strain (basLS) segments were calculated and recorded separately. The longitudinal strain measurements for each myocardial section, along with the corresponding 18‐segment bull's‐eye plots for the different patient groups, are presented in Figure 1. Strain parameters are expressed as absolute values.

##### 2.4.2.4 CMR Examination Parameters

**FIGURE 2:**
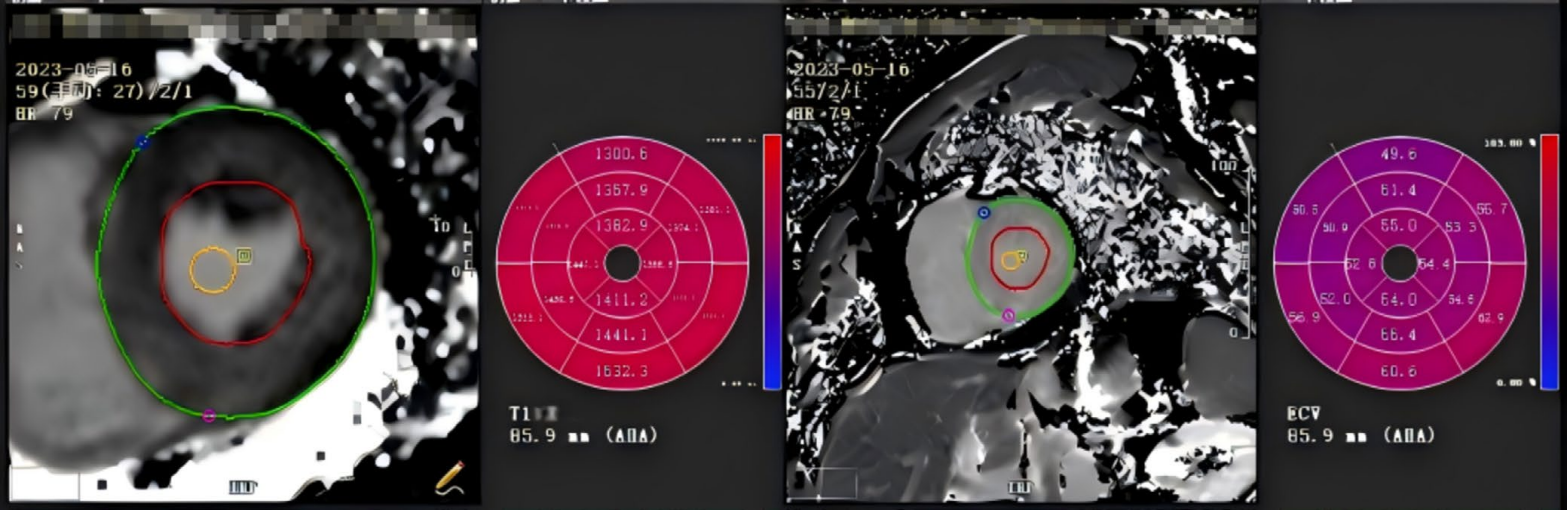
Report on myocardial native T1 and ECV parameters in the left ventricular apex, middle, and basal segments of patients with CA.

Cine‐MRI sequences and modified Look‐Locker inversion recovery sequences were collected. By using cvi42 software, the initial T1 values for the basal, middle, and apical segments of the left ventricle, as well as the overall initial T1 value of the left ventricle, were determined. Extracellular volume (ECV) was calculated using the initial T1 value before gadolinium contrast administration (Native T1) and the enhanced T1 value following contrast agent administration (Enhancement T1), based on the established formula. ECV represents the proportion of extracellular matrix volume when compared to the total myocardial tissue volume and is calculated by combining T1 values obtained before and after gadolinium contrast administration with hematocrit measurements from blood samples. The Native T1 Map for each myocardial section, along with the ECV measurement and calculation results, is depicted in Figure 2.

### 2.5 Repeatability Test

Interobserver and intraobserver variability were evaluated through the random selection of 15 patients by two physicians for the analysis of strain and magnetic resonance parameters. Variability was quantified using the intraclass correlation coefficient (ICC), which served as a measure of the rate of variation.

### 2.6 Statistical Analysis

Statistical analyses were conducted using SPSS 26.0 software. Continuous variables following a normal distribution are expressed as mean ± standard deviation ($\bar{x}\pm s$), with comparisons among multiple groups conducted using one‐way analysis of variance (ANOVA). Continuous variables that did not follow a normal distribution are presented as the median and interquartile range (M [Q1, Q3]), with comparisons among groups conducted using the Kruskal–Wallis H rank sum test. Categorical data are reported as frequency and percentage (*n*, %), and group comparisons were analyzed using the chi‐squared test. A two‐tailed test was applied to determine statistical significance, with a *p*‐value of < 0.05 being considered statistically significant.

Univariate logistic regression and backward stepwise multivariate logistic regression were used to assess the significance of variables. The receiver operating characteristic (ROC) curve was generated, and the area under the curve (AUC) was used to assess the diagnostic performance of multiparametric echocardiographic scores.

## 3 Results

### 3.1 Comparison of General Information

SBP levels in the CA group were significantly lower compared to those in the HCM and control groups, while no significant difference was observed between the HCM group and the control group. Cardiac biomarkers, including NT‐proBNP and HS‐TnT, were significantly elevated in the CA group compared to the HCM group (*p* < 0.05). The most commonly reported symptoms at the initial diagnosis in patients with CA included chest tightness, lower limb edema, and fatigue. However, the prevalence of these symptoms did not differ significantly between the CA and HCM groups (*p* > 0.05) (see Table 1).

**TABLE 1:** Comparison of general data among three groups.

Variables	CA group (*n* = 38)	HCM group (*n* = 20)	NC group (*n* = 16)	*p*
Age (years)	64.03 ± 10.52	60.85 ± 7.96	59.75 ± 4.70	0.202
Male (cases/%)	25 (65.8%)	11 (55%)	8 (50%)	0.499
BSA (m^2^)	1.65 ± 0.18	1.68 ± 0.15	1.70 ± 0.17	0.167
SBP (mmHg)	112.9 ± 21.4*^,^**	130.3 ± 12.8	131.44 ± 11.19	< 0.05
DBP (mmHg)	76.7 ± 14.7	78.8 ± 10.3	83.56 ± 8.67	0.199
NT‐proBNP (pg/mL)	1610.4 ± 1278.2**	789.2 ± 746.8	—	< 0.05
HS‐TnT (pg/mL)	51.1 ± 47.7**	17.8 ± 9.6	—	< 0.05
Chest tightness (cases/%)	16 (42.1)*	10 (50%)*	0	< 0.05
Edema in lower extremities (cases/%)	9 (23.7%)*	2 (10%)*	0	< 0.05
Fatigue (cases/%)	6 (15.8%)*	7 (35%)*	0	< 0.05

*Note:* Body surface area (m^2^) = 0.0061 × height (cm) + 0.0128 × weight (kg) − 0.1529. *p*‐values for comparison among three groups or between CA and HCM groups; *Statistically significant difference compared to the control group; **statistically significant difference compared to the HCM group.

### 3.2 Comparison of Conventional Echocardiography Parameters

The LVPWd and E/e′ values were significantly higher in the CA group compared to the HCM group, while IVSd, LVEF, and LWe′ were significantly lower in the CA group (*p* < 0.05). No significant differences were observed between the CA and HCM groups in LAD, RWT, or IVSe′. Additionally, 30 patients in the CA group had LVEF values within the range of 50% ≤ LVEF < 60%.

More patients in the CA group exhibited right ventricular wall and interatrial septum thickening, mild or more severe mitral and tricuspid valve regurgitation, and pulmonary hypertension compared to the HCM group. However, these differences did not reach statistical significance (*p* > 0.05). In contrast, the incidence of pericardial effusion was significantly higher in the CA group than in the HCM group (*p* < 0.05) (refer to Table 2).

**TABLE 2:** Analysis of conventional echocardiographic variables among the three groups.

Variables	CA group (*n* = 38)	HCM group (*n* = 20)	NC group (*n* = 16)	*p*
LAd (mm)	4.1 (3.9, 4.5)*	3.9 (3.8, 4.3)*	3.3 (3.2, 3.4)	< 0.05
LVEDd (mm)	4.5 (4.3, 4.8)	4.7 (4.3, 4.9)	4.7 (4.4, 5.0)	0.222
IVSd (mm)	1.36 ± 0.12*^,^**	1.57 ± 0.15*	0.86 ± 0.10	< 0.05
LVPWd (mm)	1.30 ± 0.13*^,^**	1.17 ± 0.14*	0.89 ± 0.09	< 0.05
RWT	0.59 ± 0.06*	0.60 ± 0.05*	0.37 ± 0.03	< 0.05
LVEF (%)	56.5 (51.8, 58.0)*^,^**	60.0 (58.3, 62.0)*	63.0 (62.0, 64.8)	< 0.05
E (cm/s)	80.1 ± 20.7	78.1 ± 17.2	73.4 ± 17.2	0.453
A (cm/s)	76.0 ± 27.9	73.5 ± 21.3	68.5 ± 12.7	0.567
E/A	1.27 ± 0.81	1.10 ± 0.4	1.2 ± 0.3	0.535
IVSe′ (cm/s)	4.3 ± 1.0*	4.8 ± 1.2*	9.5 ± 1.7	< 0.05
LWe′ (cm/s)	4.9 ± 1.6*^,^**	6.9 ± 2.0*	12.5 ± 2.8	< 0.05
E/e′	18.8 ± 7.2*^,^**	13.5 ± 5.6*	7.4 ± 2.4	< 0.05
Involvement of the right ventricular wall	10 (26.3%)	3 (15%)	—	0.509
Involvement of the interatrial septum	7 (18.4%)	0	—	0.083
Mitral regurgitation	15 (39.5%)	5 (25%)	—	0.385
Tricuspid regurgitation	9 (23.7%)	2 (10%)	—	0.538
Pericardial effusion	23 (60.5%)**	3 (15%)	—	< 0.05
Pulmonary hypertension	8 (21.1%)	5 (25%)	—	0.732

*Note:* RWT: relative wall thickness = (interventricular septal thickness + left ventricular posterior wall thickness)/left ventricular end‐diastolic diameter. *p*‐value for comparison among the three groups; *Statistically significant difference compared to the normal control group; **statistically significant difference compared to the HCM group.

### 3.3 Comparison of Left Ventricular Strain Echocardiography Parameters Among the Three Groups

**FIGURE 3:**
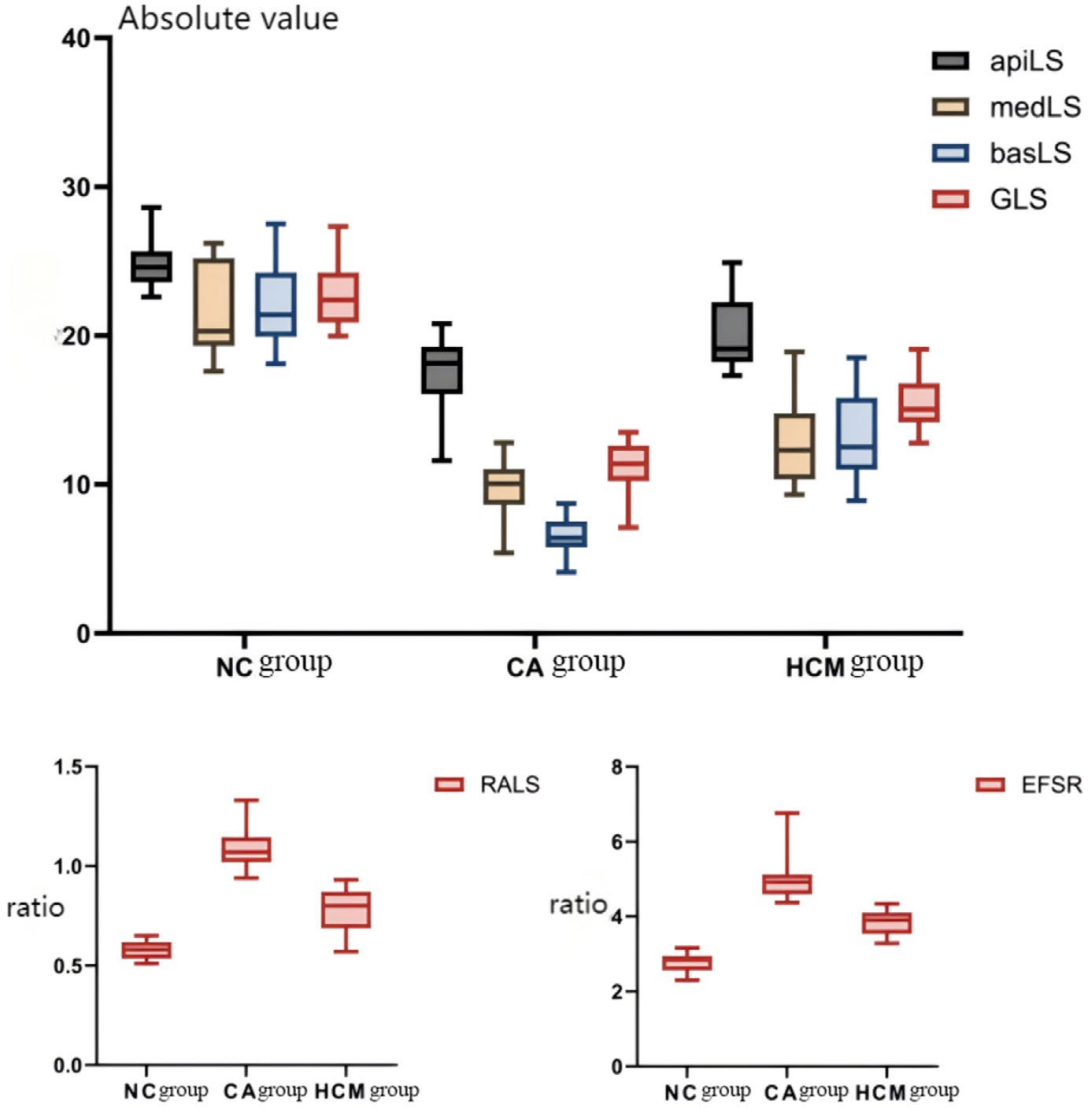
Box plot comparison of left ventricular GLS and LS, RALS, and EFSR at different segments among three groups (corresponding to Table 4).

Statistical analysis was conducted on the CA group, the HCM group, and the control group. The results indicated that the CA group demonstrated a significant reduction in apiLS, medLS, basLS, and GLS compared to both the HCM and control groups. Conversely, relative apical longitudinal sparing (RALS) and ejection fraction/longitudinal strain ratio (EFSR) exhibited a significant increasing trend in the CA group (*p* < 0.05). It is noteworthy that the HCM group did not reveal a statistically significant difference in RALS when compared to the control group (refer to Table 3 and Figure 3).

**TABLE 3:** Comparison of left ventricular strain echocardiography parameters among the three groups.

Variables	CA group (*n* = 38)	HCM group (*n* = 20)	NC group (*n* = 16)	*p*
apiLS	17.5 ± 2.2*^,^**	20.1 ± 2.3*	24.9 ± 1.9	< 0.05
medLS	9.8 ± 1.7*^,^**	12.9 ± 2.8*	21.5 ± 3.0	< 0.05
basLS	6.5 ± 1.1*^,^**	13.3 ± 2.9*	19.8 ± 2.8	< 0.05
GLS	11.3 ± 1.6*^,^**	15.4 ± 2.0*	22.8 ± 2.3	< 0.05
RALS	1.1 ± 0.19*^,^**	0.8 ± 0.11	0.6 ± 0.04	< 0.05
EFSR	4.9 ± 0.5*^,^**	3.8 ± 0.3*	2.8 ± 0.3	< 0.05

*Note:* RALS: relative apical strain = apical segment myocardial longitudinal strain/(mid segment myocardial longitudinal strain + basal segment myocardial longitudinal strain); EFSR: ejection fraction strain ratio = left ventricular ejection fraction/left ventricular global longitudinal strain. Strain parameters are all taken as absolute values. *p*‐value for comparisons among three groups or between CA and HCM groups; *Statistically significant difference compared to the normal control group; **Statistically significant difference compared to the HCM group.

### 3.4 Comparison of CMR Parameters Between CA Group and HCM Group

For CMR parameters, both the initial overall T1 value and the calculated overall ECV value were significantly higher in the CA group compared to the HCM group (*p* < 0.05) (refer to Table 4).

**TABLE 4:** Comparison of cardiac magnetic resonance parameters between the CA and HCM groups.

Variables	CA group (*n* = 38)	HCM group (*n* = 20)	*p*
Initial T1 value (ms)	1219 (1095, 1302)*	1014 (985, 1051)	< 0.05
ECV value (%)	50 ± 7*	34 ± 9	< 0.05

*Note:* ECV = (△R1_myocardium_/△R1_blood_) × (1‐Hct). *p*‐value for comparison between the CA group and the HCM group; *Statistically significant difference compared to the HCM group.

### 3.5 Construction of Multiparametric Echocardiographic Score for CA and HCM Groups

Univariate logistic regression analysis was conducted on echocardiographic variables that exhibited statistically significant differences between the CA and HCM groups based on conventional and strain echocardiographic parameters. This analysis identified six key variables: IVSd, pericardial effusion, apiLS, GLS, RALS, and EFSR. Subsequently, backward stepwise multivariate logistic regression analysis was applied to these variables, revealing that LVEF, E/e′, RALS, and EFSR could serve as diagnostic indicators for differentiating between patients diagnosed with CA and HCM (refer to Table 5).

**TABLE 5:** Results of univariate and multivariate logistic regression analysis for CA and HCM groups.

Variables	Univariate regression analysis	OR (95% CI)	Multivariate regression analysis	OR (95% CI)
	*p*		*p*	
IVSd	0.003	1.48 (1.15–1.91)	—	
PWTd	0.403	0.97 (0.91–1.12)	—	
LVEF	0.397	1.02 (0.98–1.24)	0.021	0.28 (0.10–0.83)
LWe′	0.337	0.84 (0.59–1.20)	—	
E/e′	0.103	1.03 (0.99–1.07)	0.024	1.87 (1.29–2.96)
Pericardial effusion	0.002	0.12 (0.029–0.462)	—	
apiLS	0.002	1.82 (1.24–2.67)	—	
medLS	0.496	1.10 (0.92–1.47)	—	
basLS	0.482	1.24 (0.83–1.82)	—	
GLS	0.020	0.74 (0.35–0.98)	—	
RALS	0.001	1.88 (1.23–2.78)	0.004	5.20 (1.57–17.19)
EFSR	0.001	2.27 (1.42–3.63)	0.007	4.17 (1.21–10.37)

Based on the final multivariate logistic regression analysis, an ROC curve was plotted to assess the diagnostic performance of these variables in distinguishing CA from HCM. The results revealed that the AUC for the multiparametric echocardiographic score was 0.916, which was higher than the AUC for any individual variable included in the score. Among the individual variables: RALS had an AUC of 0.837 with a cutoff value of 1.1, a sensitivity of 70%, and a specificity of 89.5%. EFSR had an AUC of 0.803 with a cutoff value of 4.5, a sensitivity of 75%, and a specificity of 86.8%. LVEF had an AUC of 0.770 with a cutoff value of 57.5%, a sensitivity of 70%, and a specificity of 68.4%. E/e′ had an AUC of 0.747 with a cutoff value of 16.6, a sensitivity of 85%, and a specificity of 57.9%.

**FIGURE 4:**
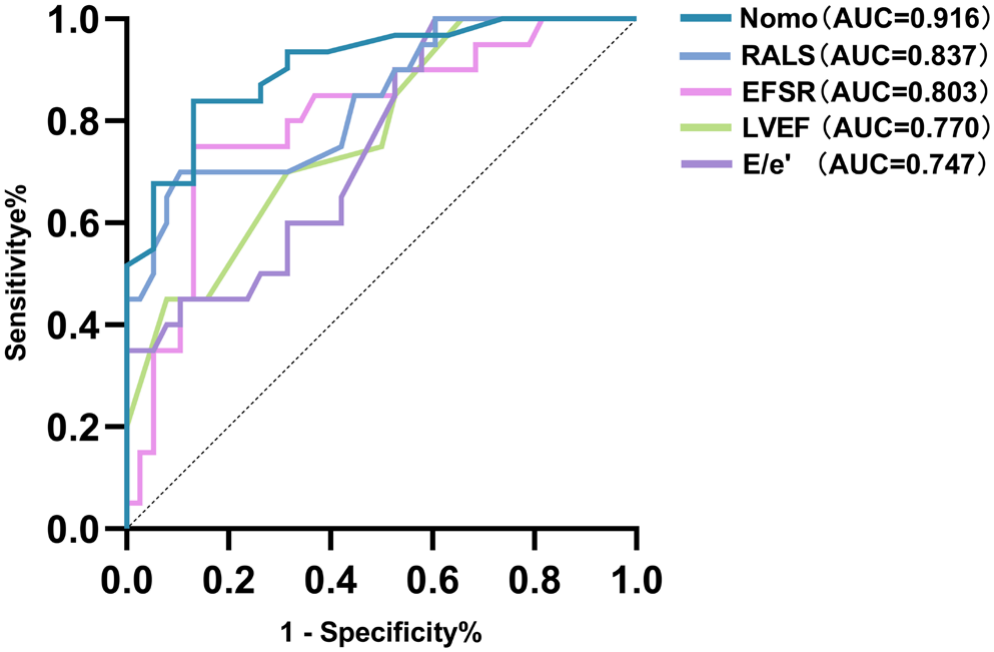
Multi‐parameter echocardiography score nomogram and ROC of various indicators.

These findings highlight the superior diagnostic efficacy of the multiparametric echocardiographic score compared to individual indicators (refer to Figure 4).

### 3.6 Correlation Analysis of Conventional/Strain Echocardiographic Parameters With ECV in Patients With CA

Pearson correlation analysis was conducted to assess the relationship between conventional and strain echocardiographic parameters and ECV values in 15 patients from the CA group who underwent concurrent CMR imaging. The analysis revealed no significant linear correlation between ECV values and LAD, LVEDD, IVSd, LVPWd, E/A ratio, LVEF, apiLS, medLS, or RALS.

**FIGURE 5:**
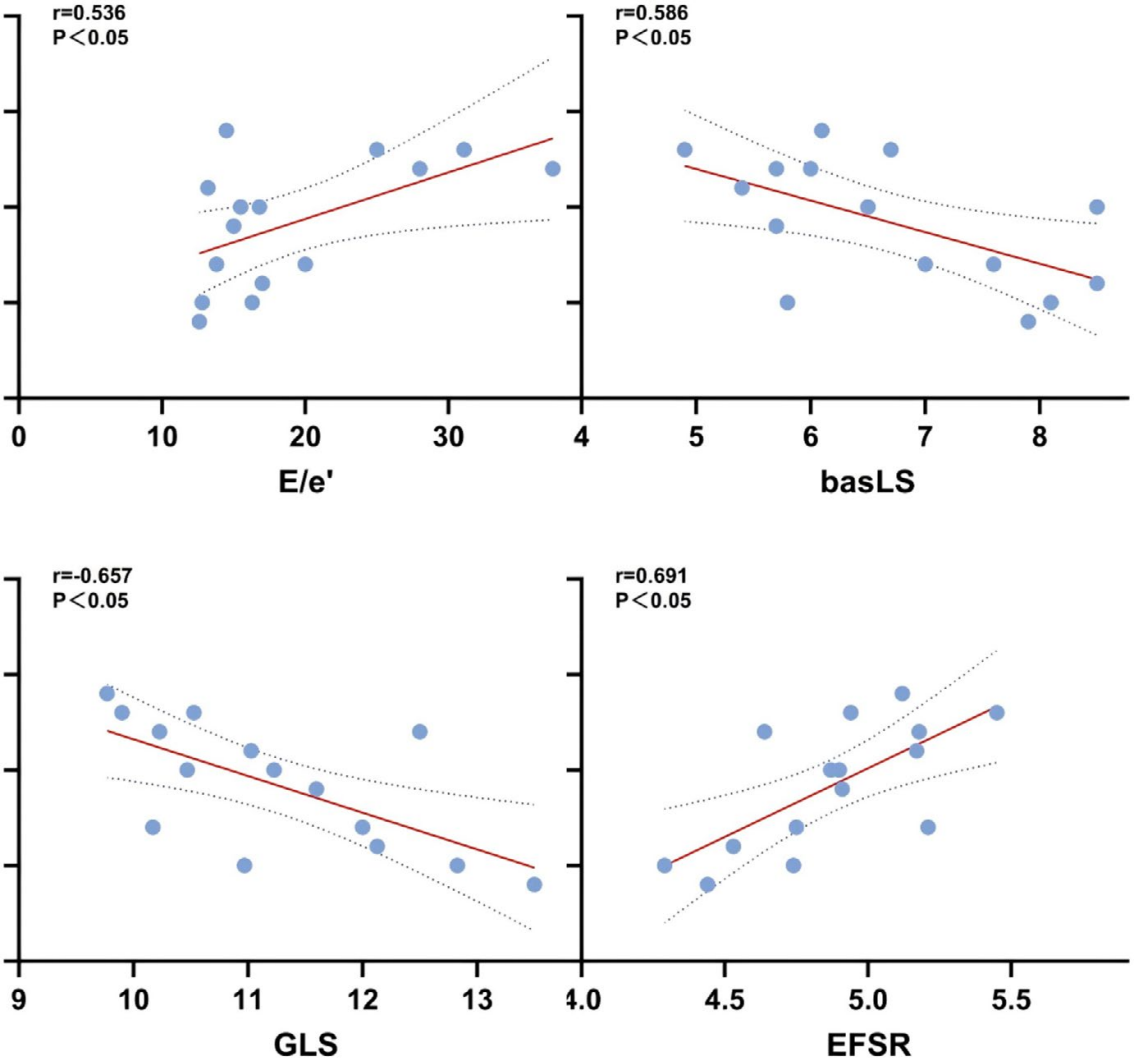
Pearson's correlation analysis between ECV values and conventional and strain echocardiography parameters (corresponding to the Table 6).

However, ECV values demonstrated a moderate positive correlation with E/e′, a moderate negative correlation with basLS, a strong negative correlation with GLS, and a strong positive correlation with the EFSR. The positive linear correlation between ECV values and EFSR indicates that GLS declines more significantly as LVEF decreases in patients with CA (refer to Table 6 and Figure 5).

**TABLE 6:** Pearson's correlation analysis between ECV values and conventional and strain echocardiography parameters.

Variables	ECV%	
	*r*	*p*
LAd	0.438	0.103
LVEDd	0.238	0.391
IVSd	0.115	0.682
LVPWd	0.274	0.322
E/A	0.332	0.227
E/e′	0.536	0.032 (< 0.05)
LVEF	−0.379	0.119
apiLS	0.298	0.280
medLS	0.04	0.875
basLS	−0.586	0.006 (< 0.05)
GLS	−0.657	0.008 (< 0.05)
RALS	0.248	0.372
EFSR	0.691	0.004 (< 0.05)

### 3.7 Repeatability Test

The ICC analyses for interobserver and intraobserver variability demonstrated good repeatability in the data measurement of left ventricular longitudinal strain parameters and ECV values (all ICC values greater than 0.75): apiLS (interobserver variability ICC: 0.911, intraobserver variability ICC: 0.934); medLS (interobserver variability ICC: 0.890, intraobserver variability ICC: 0.925); apiLS (interobserver variability ICC: 0.907, intraobserver variability ICC: 0.931).

## 4 Discussion

From this study, we discovered that the primary symptoms reported by patients with CA at the time of consultation included nonspecific manifestations such as chest tightness, shortness of breath, lower limb edema, and fatigue. The subtle and heterogeneous clinical presentation of CA often leads to diagnostic challenges, as it may be easily mistaken for other conditions. The ventricular infiltration of amyloid protein in CA results in pseudohypertrophy of the ventricular myocardium and increased myocardial stiffness, resulting in the development of restrictive cardiomyopathy. This condition is characterized by persistent restrictive pathophysiology, diastolic dysfunction, non‐dilated ventricles, and atrial enlargement (Rapezzi et al. 2022). Based on the classification of restrictive cardiomyopathy by the European Society of Cardiology, CA overlaps with HCM in diagnostic criteria, complicating their differentiation (Elliott et al. 2008). Distinguishing between CA and HCM is critical due to their differing treatment strategies and long‐term prognoses.

This study emphasized the diagnostic value of conventional echocardiographic parameters in distinguishing CA from HCM. Key differences were observed: patients with CA exhibited greater LVPWd, lower LVEF, reduced LWe′, and a significantly increased E/e′ ratio. Additionally, trace or mild pericardial effusion was more commonly observed in patients with CA. Conversely, patients with HCM exhibited greater IVSd. However, no significant difference in relative ventricular wall thickness was identified between the two groups.

The majority of patients with CA in this study presented with varying degrees of heart failure. Among them, 34 were diagnosed with HFpEF, including 30 patients with LVEF values between 50% and 60%. (Oghina et al. 2021) CA is increasingly recognized as a contributor to HFpEF, and compared to patients with HCM, the LVEF of patients with CA typically remained near normal or slightly below 60%. Previous research has indicated that an interatrial septal thickness exceeding 6 mm is a specific marker for diagnosing CA (Falk et al. 1987). However, the incidence of interatrial septum thickening in patients with CA is relatively low. In this study, no statistically significant difference in interatrial septal thickness was observed between the CA and HCM groups, indicating limited use of this parameter for diagnosing CA. However, in patients presenting with left ventricular hypertrophy and concurrent thickening of the atrial septum, clinicians should maintain a high index for a potential CA diagnosis.

Although conventional echocardiography remains a primary screening tool for CA, its sensitivity is limited. Yet, aCMQ offers enhanced precision by assessing early strain changes in myocardial segments through acoustic speckle tracking of myocardial motion, even during phases of preserved left ventricular systolic function. In this study, myocardial LS in the apical, middle, and basal segments, as well as GLS, was significantly reduced in the CA group compared to both the HCM group and the control group. The most pronounced reduction in strain was observed in the basal segment. Box plot analysis demonstrated a progressive decrease in myocardial LS from the apical to the basal segment in CA patients, a pattern not observed in patients with HCM, where no significant difference was noted between the longitudinal strain of the middle and basal segments. This characteristic strain pattern in CA, referred to as “relative apical sparing,” is marked by an increased RALS ratio and is visually represented as a “strawberry sign” on the strain bull's‐eye plot (Rapezzi and Fontana 2019; Senapati et al. 2016). A cutoff value of 1.1 for RALS was found to provide high sensitivity and specificity in distinguishing CA from HCM, which is consistent with prior findings (Phelan et al. 2012).

Pagourelias et al. introduced a novel index, the EFSR, based on the observation that patients with CA and normal EF exhibit reduced GLS (Pagourelias et al. 2017). This index demonstrated optimal discriminative ability for CA and retained strong diagnostic performance even in patients with mild ventricular hypertrophy. Their findings indicated that the increase in EFSR values in CA may primarily result from a reduction in longitudinal strain of the left ventricular myocardium. Martel et al. conducted a study including CA and HCM groups matched for maximal wall and septal wall thickness, reporting elevated EFSR values in CA patients compared to those with HCM, with a maximal AUC for CA diagnosis (Martel et al. 2023). However, these findings differ from the present study. This discrepancy could be attributed to the higher average LVEF in the population studied by Martel et al. compared to this group, as well as methodological differences in assessing LVEF, potentially influencing the discriminative diagnostic efficacy of EFSR.

With advancements in medical imaging technology, CMR imaging has emerged as a valuable tool for the diagnosis and management of CA. CMR offers significant advantages by accurately assessing the extent of amyloid protein infiltration within the myocardial interstitium, facilitated by specific sequences and its superior imaging sensitivity. Korthals et al. demonstrated that patients with CA exhibit elevated initial myocardial T1 and ECV values, which effectively reflect the degree of amyloid protein infiltration in the extracellular interstitium (Korthals et al. 2021). Furthermore, their study used CMR myocardial strain technology and identified a strong correlation between myocardial strain parameters and amyloid deposition. Specifically, the absolute value of left ventricular GLS revealed a positive linear correlation with ECV, while the absolute values of radial strain and circumferential strain exhibited negative linear correlations with ECV. These findings align with the results from this study, which also support a significant correlation between GLS and ECV values.

Boldrini et al. conducted a multicenter study involving 332 patients with AL‐CA and 339 with ATTR‐CA, identifying changes in structural and functional indices, including conventional and strain echocardiographic parameters, as the extent of cardiac amyloid infiltration increased (Boldrini et al. 2020). Their findings indicated that at low levels of myocardial infiltration, E/e′ and LS could serve as early diagnostic markers of CA. Consistently, this study also observed a moderate correlation between E/e′ and myocardial amyloid burden, as well as a strong correlation between GLS and myocardial amyloid burden.

However, RALS, which is considered highly sensitive and specific for diagnosing CA, did not demonstrate a significant correlation with ECV values. This indicates that RALS is not directly associated with the degree of myocardial amyloid infiltration, indicating no significant relationship between the extent of infiltration and relative myocardial preservation.

## 5 Conclusion

Several limitations of this study should be acknowledged: (1) This was a small‐sample, retrospective, single‐center study, with all participants recruited from a single cardiomyopathy center. Future research should involve collaboration with other cardiomyopathy centers to increase the sample size and enhance the generalizability of the findings. (2) The study was cross‐sectional and lacked long‐term follow‐up, limiting the ability to assess the prognostic use and long‐term implications of multimodal imaging in patients with CA. Prospective studies are needed to address this gap. (3) Due to the limited sample size, the study did not categorize the patients with CA into AL or ATTR subtypes. Additionally, specific cardiac radionuclide imaging for patients with ATTR‐CA was not included in the multimodal imaging evaluation. Future studies should explore the correlation between echocardiographic parameters and specific imaging findings for ATTR‐CA.

In summary, CA as an infiltrative cardiomyopathy involving multiple organ systems necessitates a multidisciplinary diagnostic and therapeutic approach. The combination of conventional and strain echocardiographic parameters provides optimal diagnostic efficacy for differentiating CA from HCM. Changes in cardiac structural and functional metrics can reflect the extent of cardiac amyloid infiltration to some degree. Implementing a comprehensive diagnostic and treatment strategy, along with early detection protocols for CA, is crucial for reducing the risk of missed or incorrect diagnoses. Such an approach ensures timely and accurate medical intervention for CA patients, ultimately improving patient outcomes.

## Author Contributions

Mei Zhu and Yong‐le Sun conceived the idea and conceptualized the study. Yong‐le Sun and Mei Zhu collected the data. Hai‐tao Yuan and Cui‐yan Wang analyzed the data. Zi‐xin Yang did the statistical analysis. Hai‐tao Yuan obtained the funding. Zi‐xin Yang and Rong‐hui Zhen drafted the manuscript, then Rong‐hui Zhen and Cui‐yan Wang reviewed the manuscript. All authors read and approved the final draft.

## Ethics Statement

This study was conducted with approval from the Ethics Committee of Shandong Provincial Hospital Affiliated to Shandong First Medical University. The approval number is SWYX: NO. 2022‐378. This study was conducted in accordance with the declaration of Helsinki. Written informed consent was obtained from all participants.

## Consent

The authors have nothing to report.

## Conflicts of Interest

The authors declare no conflicts of interest.

## Data Availability

All data generated or analyzed during this study are included in this article. Further enquiries can be directed to the corresponding author.
